# Videofluoroscopy dysphagia severity scale is predictive of subsequent remote pneumonia in dysphagia patients

**DOI:** 10.7150/ijms.76448

**Published:** 2023-02-05

**Authors:** Poyin Huang, Yi-Chiung Hsu, Chien-Hsun Li, Sun-Wung Hsieh, Kuo-Wei Lee, Kun-Han Wu, Wen-Ching Chen, Chung-Wei Lin, Chun-Hung Chen

**Affiliations:** 1 Department of Neurology, Kaohsiung Medical University Hospital, Kaohsiung Medical University, Kaohsiung, Taiwan.; 2 Department of Neurology, Kaohsiung Municipal Siaogang Hospital, Kaohsiung Medical University, Kaohsiung, Taiwan.; 3 Neuroscience Research Center, Kaohsiung Medical University, Kaohsiung, Taiwan.; 4 Department of Neurology, Faculty of Medicine, College of Medicine, Kaohsiung Medical University, Kaohsiung, Taiwan.; 5 Dysphagia Functional Reconstructive Center, Kaohsiung Municipal Siaogang Hospital, Kaohsiung Medical University, Kaohsiung, Taiwan.; 6 Multidisciplinary Swallowing Center, Kaohsiung Municipal Siaogang Hospital, Kaohsiung Medical University, Kaohsiung, Taiwan.; 7 Department of Biomedical Sciences and Engineering, National Central University, Chung-Li, Taoyuan City 320, Taiwan.; 8 Integrated Center of Healthy and Long-term Care, Kaohsiung Municipal Siaogang Hospital, Kaohsiung Medical University, Kaohsiung 812, Taiwan.; 9 Department of general medicine, Kaohsiung Medical University Hospital, Kaohsiung Medical University, Kaohsiung, Taiwan; 10 School of Medicine, College of Medicine, Kaohsiung Medical University, Kaohsiung, Taiwan.

**Keywords:** videofluoroscopy, Dysphagia Severity Scale, dysphagia, pneumonia

## Abstract

**Introduction:** Dysphagia-associated pneumonia is a critical health issue especially in the elders and stroke patients which carries a poorer prognosis. Therefore, we aim to identify methods with the potentials to predict subsequent pneumonia in dysphagia patients, which will be of great value in the prevention and early management of pneumonia.

**Methods:** One-hundred dysphagia patients were enrolled and measurements including Dysphagia Severity Scale (DSS), Functional Oral Intake Scale (FOIS), Ohkuma Questionnaire, and Eating Assessment Tool-10 (EAT-10) were assessed by either videofluoroscopy (VF), videoendoscopy (VE), or the study nurse. The patients were categorized into mild or severe groups based on each screening method. All the patients were assessed for pneumonia at 1, 3, 6, and 20 months after the examinations.

**Results:** VF-DSS (p=0.001) is the only measurement being significantly associated with subsequent pneumonia with sensitivity and specificity of 0.857 and 0.486. The Kaplan-Meier curves revealed that significant differences between the mild/severe groups start to emerge 3 months after VF-DSS (p=0.013). Cox regression models used for adjusted hazard ratio of severe VF-DSS in association with subsequent pneumonia of different timepoints after controlling the important covariates showed the following results: 3 months, p=0.026, HR=5.341, 95%CI=1.219-23.405; 6 months, p=0.015, HR=4.557, 95%CI=1.338-15.522; 20 months, p=0.004, HR=4.832, 95%CI=1.670-13.984.

**Conclusions:** Dysphagia severity evaluated by VE-DSS, VE-FOIS, VF-FOIS, Ohkuma Questionnaire, and EAT-10 is not associated with subsequent pneumonia. Only VF-DSS is associated with both short-term and long-term subsequent pneumonia. In patients with dysphagia, VF-DSS is predictive of subsequent pneumonia.

## Introduction

Dysphagia is a critical health issue that affects people around the world. It is defined as a swallowing impairment, which may lead to aspiration or penetration of the airway.^1^ Based on the site of swallowing difficulties, dysphagia can be roughly categorized as oropharyngeal dysphagia, esophageal dysphagia, or a combination of both.^2^ Due to its severity, people suffering from each type of dysphagia may develop a series of complications, which include but are not limited to pneumonia, dehydration, malnutrition, and weight loss.^1^,^3^,^4^ These patients, especially the elders and stroke patients with neurological origins, usually have poorer overall quality and length of life.^1^,^2^,^3^,^4^ A systemic review that collected literature until November 2021 revealed that the overall estimate of the global prevalence rate of oropharyngeal dysphagia was 43.8%.^5^ The increasing prevalence of dysphagia brings about a heavy burden for the social healthcare systems and economy.^3^

As to the evaluation of oropharyngeal dysphagia, Videofluoroscopy (VF) swallowing study, the gold standard survey for swallowing difficulties, is one of the most-used methods for clinicians nowadays.^3^,^4^,^6^,^7^ The patients take radiopaque materials with the instructions of the radiologists and clinicians, and are carefully observed using the X-ray equipment.^3^,^4^,^7^ Any dynamic abnormalities during the movements of swallowing will be examined in detail, which help the physicians gain insights into the clinical situations of the patients.^3^,^4^,^7^ Videoendoscopy (VE), another commonly-used tool for assessment, is also effective in the evaluation of dysphagia.^4^,^6^ It is performed by a clinician using a fiberscope to pass smoothly from the nostril, pharynx to larynx for examinations.^3^,^4^,^6^,^7^ With VE, the clinicians can easily identify the structural and motional defects during swallowing for further assessments.^3^,^4^,^6^,^7^ Aside from the above two methods, there are a variety of substitution options as assessment tools, such as history taking, physical examinations, and personal consultation.^2^,^4^ The results can be either directly utilized as the preliminary speculations of the symptoms, or the reference for further management of dysphagia in combination of miscellaneous scales.

After being clinically diagnosed and treated, most patients get relief from oropharyngeal dysphagia. However, still a proportion of patients have limited improvement in symptoms, which may be accompanied with more severe complications afterwards. Among all the complications, aspiration pneumonia is one of the most severe events of oropharyngeal dysphagia, especially in post-stroke patients.^6^ The severity and the biosocial impact of pneumonia not only lead to an increase in mortality and morbidity, but also an estimated $4.4 billion of annual health-care cost in the United States, which deserves our attention.^6^,^7^,^8^,^9^,^10^ Recently, there are emerging researches advocating the close relationship between oropharyngeal dysphagia and aspiration pneumonia. However, it remains unknown how to accurately predict the occurrence of pneumonia by early assessments in the high-risked dysphagia patients. In addition, there are limited studies discussing the course of pneumonia development after swallowing examinations with a long-term follow up. Therefore, we aim to identify scales or methods with the potentials to predict subsequent pneumonia, as well as the occurrence of pneumonia at multiple timepoints based on the severity of oropharyngeal dysphagia. This will be of great value in the prevention and early management of pneumonia for clinicians.

## Materials and Methods

### Study population

A total of 100 patients were enrolled in this study. The patients recruited suffered from subjective dysphagia caused by cerebrovascular diseases, normal aging, and other diseases. These patients took four different examinations as a set for further assessments during 2019 and 2020. Based on the results of each dysphagia test, the patients were further categorized either as the “mild group” or the “severe group” for further comparisons and analyses.

After the examinations were completed, we had closely followed up the patients' general health conditions, which mainly focused on the development of pneumonia in the next 20 months. The patients were reassessed after the swallowing examinations at 1 month (4 weeks), 3 months (12 weeks), 6 months (24 weeks), and 20 months, respectively, for later analysis.

### Dysphagia Evaluation

To assess the presence and severity of dysphagia, the patients underwent as many as four screening tests by either VF, VE or the study nurse for further evaluation and analysis: Dysphagia Severity Scale (DSS), Functional Oral Intake Scale (FOIS), Ohkuma Questionnaire, and Eating Assessment Tool-10 (EAT-10). VF was performed and examined by one trained radiologist, VE by two trained neurologists, other questionnaires and scales by one trained study nurse to minimize the inter-operator bias. These four functional outcome measurements are introduced as follows (supplements 1-4):

1. DSS, which may be performed by physicians with either VF or VE and by study nurse's judgement, serves as a useful instrument to determine the severity of dysphagia. According to the condition of aspiration, seven levels are divided. Those ranked from “level 1” to “level 4'' were categorized into the “choking/aspiration group (severe group)”, otherwise the “without choking/aspiration group (mild group)” if ranked from “level 5” to “level 7”.

2. FOIS, which is performed by physicians with either VF or VE, reflects the patients' functional oral intake condition. According to the need of tube supplements and oral intake condition, seven levels are divided. Those ranked from “level 1” to “level 3'' were categorized into the “tube-dependent group (severe group)”, otherwise the “tube-independent group (mild group)” if ranked from “level 4” to “level 7”.

3. Ohkuma Questionnaire, being a convenient and validated measurement to assess the overall swallowing condition over the past three months, provides fifteen comprehensible questions for evaluation.^11^ If more than one answer of the questions is classified as severe symptoms, the patients will be classified as the “severe group”, otherwise the “mild group”.

4. EAT-10, a well-recognized questionnaire for dysphagia evaluation, offers ten common clinical situations in relation to patients' swallowing difficulties over the past three months. The answer of each question is rated based on the severity of the symptoms from “0” (no problem) to “4” (severe problem). If the sum of points of all questions is more than three, the patient will be categorized as the “severe group”, otherwise the “mild group”.

### Data Collection & Statistical Analysis

This study was approved by the Institutional Review Board of our hospital, and the number of IRB is KMUHIRB-F(II)-20190133. Written informed consent was obtained from all patients or their relatives before participating in this study. We reviewed the medical records of the patients, and prospectively collected the data for later analysis. The basic characteristics including age, sex, and clinical characteristics of swallowing examinations, as well as the presence and timepoints of pneumonia development were recorded. Primary etiologies of dysphagia and underlying diseases were also recorded.

As to the data processing, we first categorized the patients into the “mild group” and the “severe group”. Fisher's exact test was performed to identify the association with subsequent pneumonia development, based on the “mild group” and the “severe group” differentiated by VF-DSS, VE-DSS, Nursing-DSS, VF-FOIS, VE-FOIS, Ohkuma questionnaire, and EAT-10, in the first 20 months after these swallowing examinations were performed. The ROC (receiver operating characteristic) curve was used for the tool with significant association with subsequent pneumonia to further identify its sensitivity and specificity.

Secondly, for the tool which was significantly associated with subsequent pneumonia in the first step, we used Fisher's exact test to identify from which point of time after the swallowing examinations would mark a significant difference in pneumonia development between the two different dysphagia groups. The timepoints we observed included 1 month (4 weeks), 3 months (12 weeks), 6 months (24 weeks), and 20 months after the swallowing examinations.

Lastly, for the tool which was significantly associated with subsequent pneumonia in the first step, we used the Kaplan-Meier curve for the follow-up occurrence of pneumonia between the two dysphagia groups, which lasted from 1 month (4 weeks), 3 months (12 weeks), 6 months (24 weeks), to 20 months. Cox regression models were also used to verify the significance of the variables and the adjusted hazard ratios. Since all the covariates except the tool of interest showed no significant association in univariate analysis, only the tool of interest and the etiology of dysphagia, which is considered an important confound, were put into the model. All of the data were processed using IBM SPSS Statistics Version 22.

## Results

The basic characteristics of the 100 dysphagia patients are presented as Table 1. Primary causes of dysphagia included stroke, aging-related, and other diseases. For aging-related dysphagia, other possible etiologies of dysphagia were excluded, and aging was the only identified etiology of dysphagia after surveys. Other causes of dysphagia included intracranial tumor (4%, N=4), head and neck cancer (4%, N=4), Parkinson disease (2%, N=2), encephalitis (1%, N=1), and amyotrophic lateral sclerosis (1%, N=1). There is no significant difference in both groups in the baseline features.

We analyzed the relationship between dysphagia groups and pneumonia occurrence after a 20-month follow-up in the four scales (Table 2). The Fisher's exact test reveals that VF-DSS (p=0.001), other than VE-DSS (p=0.646), Nursing-DSS (p=0.599), VF-FOIS (p=0.262), VE-FOIS (p=0.286), Ohkuma Questionnaire (p=0.569), and EAT-10 (p=0.633), is the only measurement with the initial results significantly associated with later pneumonia occurrence. The subgroup analysis was performed on the basis of VF-DSS results.

In VF-DSS, the p-value analyzed between the “severe group” and the “mild group” was 0.001, indicating that subsequent pneumonia development was significantly associated with the severity of dysphagia examined by this tool. Since it could possibly serve as an effective tool in predicting the development of pneumonia after a 20-month follow-up, we further calculated the sensitivity and specificity of pneumonia prediction by ROC curve. The sensitivity and specificity of VF-DSS are 0.857 and 0.486 when using 1-4 aspiration versus 5-7 non-aspiration as the cutoff point, respectively. [Cutoff point=4.5; AUC (area under curve) =0.697]

**Figure 1:**
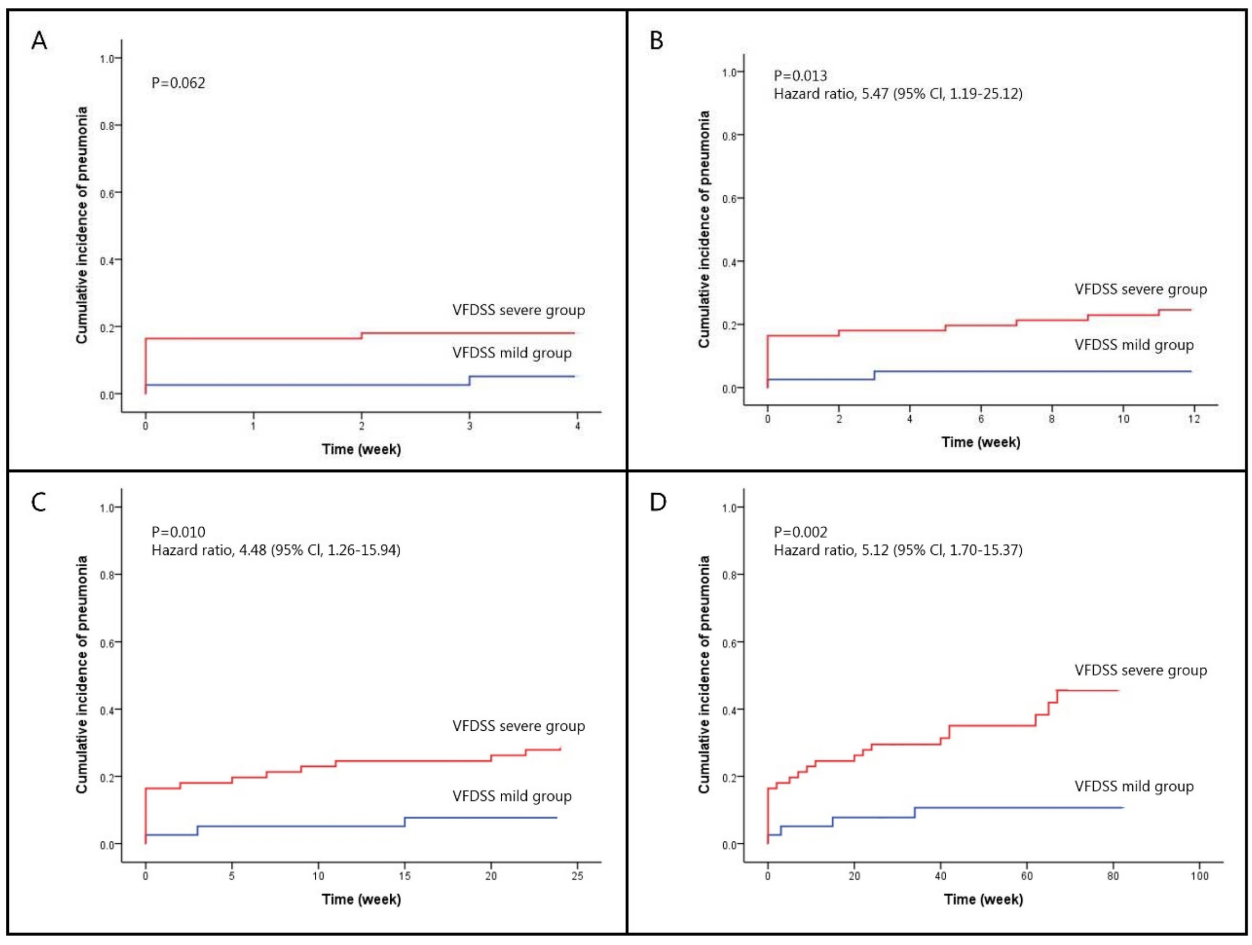
Kaplan-Meier curve of pneumonia occurrence compared between the mild and severe VF-DSS groups after different follow-up durations. (A) After a 1-month (4 weeks) follow-up, there was no significant difference (p=0.062) in pneumonia occurrence between the mild and severe VF-DSS groups. (B) After a 3-month (12 weeks) follow-up, pneumonia occurrence in the severe VF-DSS group was significantly greater (hazard ratio=5.47, 95%CI=[1.19, 25.12]; p=0.013) than in the mild VF-DSS group. (C) After a 6-month (24 weeks) follow-up, pneumonia occurrence in the severe VF-DSS group was significantly greater (hazard ratio=4.48, 95%CI=[1.26, 15.94]; p=0.010) than in the mild VF-DSS group. (D) After a 20-month (80 weeks) follow-up, pneumonia occurrence in the severe VF-DSS group is significantly greater (hazard ratio=5.12, 95%CI=[1.70, 15.37]; p=0.002) than in the mild VF-DSS group.

In addition, we analyzed timepoints of 1 month, 3 months, 6 months, and 20 months, in which pneumonia ever occurred after VF-DSS tests (Table 3). Fisher's exact tests show that in both VF-DSS groups, there is no significant difference in pneumonia development in the first month (p=0.073). Nonetheless, significant differences between the two groups start to emerge 3 months after VF-DSS tests (p=0.013); furthermore, the occurrence of pneumonia that ever happened in 6 months (p=0.011) and 20 months (p=0.001) became more significantly different. The Kaplan-Meier curves of the two VF-DSS dysphagia groups with different follow-up durations are presented as Figure 1, which lasted from 1 month (log rank, p=0.062), 3 months (log rank, p=0.013), 6 months (log rank, p=0.010), and 20 months (log rank, p=0.002). Cox regression models used for adjusted hazard ratio of severe VF-DSS in association with subsequent pneumonia of different timepoints after controlling the important covariates (Table 4) showed the following results: 3 months, p=0.026, HR=5.341, 95%CI=1.219-23.405; 6 months, p=0.015, HR=4.557, 95%CI=1.338-15.522; 20 months, p=0.004, HR=4.832, 95%CI=1.670-13.984. When using age, gender, diabetes mellitus, hypertension, hyperlipidemia, and etiologies as covariates in COX models, forward and backward variable selection procedure generated the similar results that only VF-DSS and etiologies remained in the models.

## Discussion

This study aims at figuring out the suitable methods of dysphagia evaluation for accurate pneumonia prediction. The results demonstrate that only VF-DSS shows a significant difference (p=0.001) in the follow-up pneumonia occurrences based on the initial severity of dysphagia, which is as early as 3 months (12 weeks) after the dysphagia evaluation. Up to 28% of dysphagia patients in our study had experienced pneumonia within the subsequent 20 months after the dysphagia assessments. Regarding dysphagia-associated pneumonias, it is widely-accepted that aspiration is of the utmost importance.^12^ Being one of the most common pneumonia types, aspiration pneumonia, however, is not the only type of pneumonia that causes critical problems of dysphagia patients. In fact, several studies have further recognized that it is difficult to distinguish aspiration pneumonia from other aspiration syndromes, CAPs and HAPs (hospital-acquired pneumonias) since that the pneumonia types share similarities during their clinical courses.^13^,^14^ In this study, we therefore loosely defined the “dysphagia-associated pneumonias”, which was not limited to aspiration pneumonia for better evaluations and analyses. The results are even more reasonable and close to the clinical situations in the real-world practice.

As previously mentioned, being a critical complication of dysphagia, pneumonia is essential to be early predicted. Therefore, early assessments of dysphagia may help the clinicians identify the risks of future pneumonia occurrences in the patients. As to the assessment tools for dysphagia, many literature reports had focused on certain bedside screening tools, which was thoroughly discussed in a systematic review article.^1^ Aside from our study, however, there is no previous research using as many dysphagia screening methods as ours in one prospective research to figure out which of them may be the suitable predictors for pneumonia. In this study, the screening methods we used were proven to possess reliability and validity for dysphagia detection.^16^,^17^,^18^,^19^,^20^,^21^,^22^ Ohkuma questionnaire and EAT-10, for instance, are questionnaire-based tools which are evaluated by personal judgements. Ohkuma questionnaire, a 15 question-based test focusing more on the dysphagia-related life experience of the patients, is relatively a more comprehensive questionnaire compared to EAT-10, which is a 10 question-based test originating from the personal experience of dysphagia. DSS and FOIS are the two mostly-used scales for dysphagia assessments since they grade the patients more specifically based on their clinical swallowing situations. Both of them can be performed not only by the physicians with either VF or VE, but also the nurse staff's judgments.^22^ All of the above are commonly-used methods in clinical practice.

Despite the advantages of these bedside rating scales, a recent systematic review by O'Horo et al. concluded that no bedside screening protocol was shown to provide adequate predictive value for presence of aspiration, except for VF- or VE-based maneuvers. This is close to our knowledge that both of the tools are more adequate in detecting aspiration events. In clinical practice, during the process, whether the contrast enhancements enter the airway or not can be precisely detected by VF and VE. In other words, besides active dysphagia problems, even silent aspiration can be clearly observed and recognized, making it more accurate to identify the patients with severe symptoms or not. Based on the statement, we furthermore figured out that none of the bedside screening methods possesses a statistically significant association with subsequent pneumonia, except VF-DSS (p=0.002). In addition, in VF-DSS, only the severe group is highly associated with the occurrence of pneumonia, which is not only in the short-term (3 months) but also in the long-term (20 months) follow-up duration (Table 3) (Figure 1). The findings above may explain how VF-DSS plays a crucial role in the connection between dysphagia and aspiration, as well as its potential application in predicting the future pneumonia events.

Being an assessment tool with high accuracy, VF has been viewed as the traditional gold standard of dysphagia screening for a long time.^23^ VE, as a relatively novel instrument, has also been promoted by many researchers since the 1990s.^23^ Recently, the comparisons between VF and VE have been widely reported in the literature.^23^,^24^,^25^,^26^ Studies have claimed that VE also serves as an effective instrument for dysphagia evaluations and outcomes. A research conducted by Wu and his colleagues mentioned that VE was more sensitive in detecting some risky features, including pharyngeal stasis, laryngeal penetration, aspiration, effective cough reflex, and velopharyngeal incompetence.^23^ They deemed VE more accessible and efficient than VF. In a prospective research of Giraldo-Cadavid et al., they also agreed that VE was better than VF in detecting aspiration, penetration, and residues.^24^ Based on the above, it is quite credible that VE may not be inferior to VF in clinical practice. Given that both VF and VE have the advantages in evaluating dysphagia and detecting certain outcomes from previous studies, they are the main focus of our study. However, only VF was found to be predictive of future pneumonia while VE was not. This result could serve as a reference for future relative researches. It is worth mentioning that VF can be combined with many screening scales including PAS (Penetration-Aspiration Scale) and FDS (Functional Dysphagia Scale) which have been utilized in many studies. Both VF-PAS and VF-FDS were proven to serve as sensitive and specific methods for quantifying the severity of dysphagia.^27^,^28^,^29^,^30^ However, neither of them has the diagnostic value in predicting subsequent pneumonia.^28^ Regarding our study, the high sensitivity (0.857) and low specificity (0.486) of VF-DSS, which is shown in the ROC curve analysis, implies that using VF-DSS for the subsequent pneumonia prediction is valuable when the patients were categorized as mild but rather unuseful when the patients were categorized as severe since patients with mild dysphagia were unlikely to develop subsequent pneumonia. Last but not least, in clinical practice, the invasiveness, cost, and availability of a tool should be taken into consideration. Bedside assessments do have their role in assessing the risk of aspiration and, VF, just like most image exams has the limitations of being invasive and equipment-dependent.

There are some limitations in our study. First of all, the small sample size of this study may have an impact on the results since the lack of statistical significance of tools other than VF-DSS may be caused by the insufficiency of power. Future studies with more samples are warranted to verify our points of discussion. Secondly, the etiologies of dysphagia were heterogeneous in this study. However, there was no significant difference in etiologies between the two VF-DSS groups. Furthermore, the Cox regression model showed that the etiologies of dysphagia are not associated with subsequent pneumonia, thus diminishing the impact of heterogeneity. Thirdly, the baseline functional severity and cognitive status of the recruited patients were not investigated. Both of these parameters do have an impact on swallowing and should be taken into consideration in future studies.

In conclusion, dysphagia severity evaluated by VE-DSS, VE-FOIS, VF-FOIS, Ohkuma Questionnaire, and EAT-10 is not associated with subsequent pneumonia. Only VF-DSS is associated with both short-term and long-term subsequent pneumonia. In patients with dysphagia, VF-DSS is predictive of subsequent pneumonia.

## Supplementary Material

Supplementary material.

## Figures and Tables

**Table 1:** Basic characteristics of the patients with dysphagia

Basic Characteristics	Total patients	†VF-DSS		
	(N=100)	†Mild (N=39)	†Severe (N=61)	P-value
**Age (years)**	63.3 ± 13.8	62.5 ± 11.6	63.6 ± 15.3	0.670
**Gender**				
Male	70.0% (N=70)	59.0% (N=23)	77.0% (N=47)	0.074
Female	30.0% (N=30)	41.0% (N=16)	23.0% (N=14)	
**Primary cause of dysphagia**				
Ischemic stroke	61% (N=61)	64.1% (N=25)	59.0% (N=36)	0.893
Hemorrhage stroke	11% (N=11)	7.7% (N=3)	13.1% (N=8)	
Aging	13% (N=13)	12.8% (N=5)	13.1% (N=8)	
Others	15% (N=15)	15.4% (N=6)	14.8% (N=9)	
**Specific disease history**				
Diabetes mellitus	46.0% (N=46)	43.6% (N=17)	47.5% (N=29)	0.523
Hypertension	74.0% (N=74)	79.5% (N=31)	70.5% (N=43)	0.580
Dyslipidemia	51.0% (N=51)	59.0% (N=23)	45.9% (N=28)	0.384

* Data are presented either as percentage (n %) or mean ± standard deviation (SD). # P-values are calculated using independent t-tests and Fisher's exact tests. † VF-DSS=(Videofluoroscopy) *Dysphagia Severity Scale*. In VF-DSS, patients ranked from “level 1” to “level 4'' are classified as the “severe group”, otherwise the “mild group” if ranked from “level 5” to “level 7''.

**Table 2:** Pneumonia developing in follow-up 20 months after different rating scale assessment in the patients with dysphagia

Rating scale	Total patients	Pneumonia in follow-up 20 months		P-value
		Yes	No	
† **VF-DSS**				0.001
Severe (Level 1-4)	61% (N=61)	39% (N=24)	61% (N=37)	
Mild (Level 5-7)	39% (N=39)	10% (N=4)	90% (N=35)	
† **VF-FOIS**				
Severe (Level 1-3)	9% (N=9)	44% (N=4)	56% (N=5)	0.262
Mild (Level 4-7)	91% (N=91)	26% (N=24)	74% (N=67)	
† **VE-DSS**				
Severe (Level 1-4)	47% (N=42)	33% (N=14)	67% (N=28)	0.646
Mild (Level 5-7)	52% (N=47)	28% (N=13)	72% (N=34)	
† **VE-FOIS**				
Severe (Level 1-3)	22% (N=20)	40% (N=8)	60% (N=12)	0.286
Mild (Level 4-7)	78% (N=69)	28% (N=19)	72% (N=50)	
†** Nursing-DSS**				0.599
Severe (Level 1-4)	56% (N=44)	27% (N=12)	73% (N=32)	
Mild (Level 5-7)	44% (N=34)	21% (N=7)	79% (N=27)	
†** Ohkuma**				
Severe (severe symptoms ≥1)	80% (N=72)	32% (N=23)	68% (N=49)	0.569
Mild (severe symptoms =0)	20% (N=18)	22% (N=4)	78% (N=14)	
† **EAT-10**				0.633
Severe (≥ 3 points)	71% (N=71)	30% (N=21)	70% (N=50)	
Mild (< 3 points)	29% (N=29)	24% (N=7)	76% (N=22)	

* Data are presented as *percentage (n %)* # In 100 patients we enrolled, not everyone has undergone all rating scale assessments, resulting in unequal numbers of patients. # P-values are calculated using Fisher's exact tests. † VF-DSS= (Videofluoroscopy) Dysphagia Severity Scale; VF-FOIS= (Videofluoroscopy) Functional Oral Intake Scale; VE-DSS= (Videoencoscopy) Dysphagia Severity Scale; VE-FOIS= (Videoencoscopy) Functional Oral Intake Scale; Nursing-DSS= Dysphagia Severity Scale assessed by nurse; Ohkuma= Ohkuma questionnaire; EAT-10= 10-item Eating Assessment tool

**Table 3:** Pneumonia developing after VF-DSS assessment in the patients with dysphagia.

Follow-Up Duration	Total patients	†VF-DSS		
	(N=100)	†Mild (N=39)	†Severe (N=61)	P-value
1 month (4 weeks)	12.7% (N=13)	5.1% (N=2)	18.0% (N=11)	0.073
3 months (12 weeks)	16.7% (N=17)	5.1% (N=2)	24.6% (N=15)	0.013
6 months (24 weeks)	20.6% (N=21)	7.7% (N=3)	29.5% (N=18)	0.011
20 months	27.5% (N=28)	10.3% (N=4)	39.3% (N=24)	0.001

* Data are presented as *percentage (n %)*. # P-values are calculated using Fisher's exact tests. † VF-DSS=(Videofluoroscopy) *Dysphagia Severity Scale*. In VF-DSS, patients ranked from “level 1” to “level 4'' are classified as the “severe group”, otherwise the “mild group” if ranked from “level 5” to “level 7''.

**Table 4:** Cox regression of 3, 6, and 20 months follow-up pneumonia occurrence adjusted with variables.

Independent variable	Pneumonia in follow-up 3 months			Pneumonia in follow-up 6 months			Pneumonia in follow-up 20 months		
	Hazard ratio	95% CI	P-value	Hazard ratio	95% CI	P-value	Hazard ratio	95% CI	P-value
†VF-DSS (severe group)	5.341	1.219-23.405	0.026	4.557	1.338-15.522	0.015	4.832	1.670-13.984	0.004
Etiology of dysphagia									
Ischemic stroke			0.274			0.062			0.185
Hemorrhagic stroke	0.588	0.073-4.709	0.617	0.522	0.066-4.126	0.538	0.316	0.042-2.389	0.265
Aging-related	1.866	0.494-7.041	0.357	2.908	0.972-8.694	0.056	1.950	0.704-5.402	0.199
Others	2.637	0.862-8.068	0.089	3.049	1.082-8.595	0.035	1.983	0.769-5.113	0.157

† VF-DSS= (Videofluoroscopy) Dysphagia Severity Scale. In VF-DSS, patients ranked from “level 1” to “level 4'' are classified as the “severe group”, otherwise the “mild group” if ranked from “level 5” to “level 7''.
