# Supplementary material for: Videofluoroscopy dysphagia severity scale is predictive of subsequent remote pneumonia in dysphagia patients
Source: Int J Med Sci. 2023 Feb 5;20(3):429–36. doi: 10.7150/ijms.76448 (PMC9969506; doi:10.7150/ijms.76448)
Supplement: Supplementary file 1 — Supplementary material. [file ijmsv20p0429s1.pdf]

Supplement 1. *Dysphagia Severity Scale* (DSS).

Level	Description
1	Saliva aspiration
2	Food aspiration
3	Water aspiration
4	Occasional aspiration
5	Oral problem
6	Minimum problem
7	Within normal limits

* In DSS, those ranked from “level 1” to “level 4” are categorized into the “choking/aspiration group (severe group)”.

* In DSS, those ranked from “level 5” to “level 7” are categorized into the “without choking/aspiration group (mild group)”.

Supplement 2. *Functional Oral Intake Scale* (FOIS).

Level	Description
1	No oral intake
2	Tube dependent with minimal/inconsistent oral intake
3	Tube supplements with consistent oral intake
4	Total oral intake of a single consistency
5	Total oral intake of multiple consistencies requiring special preparation
6	Total oral intake with no special preparation, but must avoid specific foods or liquid items
7	Total oral intake with no restrictions

* In FOIS, those ranked from “level 1” to “level 3’’ are categorized into the “tube-dependent group (severe group)”.

* In FOIS, those ranked from “level 4” to “level 7’’ are categorized into the “tube-independent group (mild group)”.

Supplement 3. *Ohkuma Questionnaire.*

Question	1	2	3
1. Have you ever been diagnosed with pneumonia?	Many times	Once	No
2. Do you feel you are becoming thin?	Obviously	Slightly	No
3. Do you ever have difficulty when you swallow?	Many times	Sometimes	No
4. Do you ever choke during a meal?	Many times	Sometimes	No
5. Do you ever choke when swallowing liquids?	Many times	Sometimes	No
6. Do you ever have difficulty with coughing up phlegm during or after a meal?	Many times	Sometimes	No
7. Do you ever have the feeling that food is getting stuck in your throat?	Many times	Sometimes	No
8. Does it take you longer to eat a meal than before?	Obviously	Slightly	No
9. Do you feel that it is getting difficult to eat solid foods?	Obviously	Slightly	No
10. Do you ever drop food from your mouth?	Many times	Sometimes	No
11. Do you ever have the feeling that food is remaining in your mouth?	Many times	Sometimes	No
12. Do you ever have the feeling of food or liquid going up into your throat from your stomach?	Many times	Sometimes	No
13. Do you ever have the feeling that food is getting stuck in your esophagus?	Many times	Sometimes	No
14. Do you ever have difficulty sleeping because of coughing during the night?	Many times	Sometimes	No
15. Do you feel that your voice is getting hoarse?	Obviously	Slightly	No

* In *Ohkuma Questionnaire*, if more than one answer of the questions is rated “1”, the patient is categorized as the “severe group”; otherwise the “mild group”.

Supplement 4. *Eating Assessment Tool-10* (EAT-10).

Question	0=No problem			4=Severe problem	
1. My swallowing problem has caused me to lose weight.	0	1	2	3	4
2. My swallowing problem interferes with my ability to go out for meals.	0	1	2	3	4
3. Swallowing liquids takes extra effort.	0	1	2	3	4
4. Swallowing solids takes extra effort.	0	1	2	3	4
5. Swallowing pills takes extra effort.	0	1	2	3	4
6. Swallowing is painful.	0	1	2	3	4
7. The pleasure of eating is affected by my swallowing.	0	1	2	3	4
8. When I swallow food sticks in my throat.	0	1	2	3	4
9. I cough when I eat.	0	1	2	3	4
10. Swallowing is stressful.	0	1	2	3	4

* In EAT-10, if the sum of points of all questions is more than “3”, the patient is categorizes as the “severe group”; otherwise the “mild group”.
